# NiCo nanoalloy encapsulated in graphene layers for improving hydrogen storage properties of LiAlH_4_

**DOI:** 10.1038/srep27429

**Published:** 2016-06-07

**Authors:** Chengli Jiao, Lixian Sun, Fen Xu, Shu-Sheng Liu, Jian Zhang, Xia Jiang, Lini Yang

**Affiliations:** 1 Key Laboratory of Biobased Materials, Qingdao Institute of Bioenergy and Bioprocess Technology, Chinese Academy of Sciences, No. 189 Songling Road, Qingdao 266101, P. R. China; 2 Guangxi Key Laboratory of Information Materials & Guangxi Collaborative Innovation Center of Structure and Property for New Energy and Materials, School of Material Science and Engineering, Guilin University of Electronic Technology, Guilin 541004, P. R. China; 3 Dalian Institute of Chemical Physics, Chinese Academy of Sciences, 457 Zhongshan Road, Dalian 116023, P. R. China; 4 INAMORI Frontier Research Center, Kyushu University, Nishi-ku, Fukuoka 8190395, Japan; 5 College of Chemistry, Liaoning University, Shenyang 110036, P. R. China

## Abstract

NiCo nanoalloy (4–6 nm) encapsulated in grapheme layers (NiCo@G) has been prepared by thermolysis of a 3D bimetallic complex CoCo[Ni(EDTA)]_2_·4H_2_O and successfully employed as a catalyst to improve the dehydrogenation performances of LiAlH_4_ by solid ball-milling. NiCo@G presents a superior catalytic effect on the dehydrogenation of LiAlH_4_. For LiAlH_4_ doped with 1 wt% NiCo@G (LiAlH_4_-1 wt% NiCo@G), the onset dehydrogenation temperature of LiAlH_4_ is as low as 43 °C, which is 109 °C lower than that of pristine LiAlH_4_. 7.3 wt% of hydrogen can be released from LiAlH_4_-1 wt% NiCo@G at 150 °C within 60 min. The activation energies of LiAlH_4_ dehydrogenation are extremely reduced by 1 wt% NiCo@G doping.

Hydrogen storage is one of the most critical issues for fuel cell vehicular applications. Solid hydrogen storage materials such as carbon materials, MOFs, metal hydrides and complex hydrides have received significant attention as the safest and most effective storage media^1^,^2^,^3^,^4^,^5^,^6^,^7^,^8^,^9^,^10^. Among them, lithium alanate (LiAlH_4_) is considered as one of the most promising hydrogen storage materials due to its high hydrogen storage capacity of 10.5 wt%. In the last two decades, LiAlH_4_ has received particular attention aiming at reducing the operation temperature to meet the DOE criteria because it can release a total amount of 7.9 wt% of hydrogen in two steps (eqs 1 and 2) below a relatively low temperature, e.g. 220 °C.


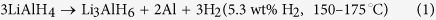



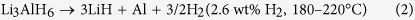


Different methods have been explored for decrease of the dehydrogenation temperature of LiAlH_4_, including particle size reduction by ball milling^11^, synthesis of multi-hydride composites^12^, and doping with catalysts^13^,^14^. Among these methods, doping with catalysts is considered as an effective approach for the dehydrogenation of LiAlH_4_. Various catalysts have been investigated, such as Ti, Fe, Ni, V, Al, Al_3_Ti, TiF_3_, TiCl_4_, TiCl_3_, NiCl_2_, VCl_3_, AlCl_3_, FeCl_3_, TiCl_3_·1/3AlCl_3_, TiH_2_, NiFe_2_O_4_, carbon nanotube and so on^15^,^16^,^17^,^18^,^19^. Ni based catalysts have been widely explored. Zheng *et al.*^20^ doped LiAlH_4_ with Ni to reduce the temperature of the first dehydrogenation step by approximately 10–15 °C, with a great expense of the hydrogen storage capacity. Yuan *et al.*^21^ prepared 2 mol% NiCo_2_O_4_ nanorod doped LiAlH_4_, which can release 6.47 wt% of hydrogen at 150 °C within 150 min. Nevertheless, most of the systems present the disadvantage of loss of the overall hydrogen storage capacity, due to either a hydrogen release during the ball milling process or the large additional weight of the catalysts. Thus, it is desirable to develop an effective catalyst for the dehydrogenation of LiAlH_4_.

Recently, metal/carbon composites has been developed for the dehydrogenation of LiAlH_4_, including Co-decorated MWCNTs^22^, Co@C^23^, and Ni-containing mesoporous carbon scaffold (Ni-MCS)^24^. In the last two decades, bimetallic nanoparticles have received great interest for catalysis and electrocatalysis, due to their higher activity and selectivity than monometallic nanoparticles as a result of “synergistic effects”^25^,^26^,^27^,^28^. In addition, graphene more effectively improves the dehydrogenation behavior of LiAlH_4_ than C_60_, carbon nanotubes, and graphite^29^. In this study, we used NiCo nano alloy encapsulated in graphene layers (NiCo@G) as a catalyst and investigated the effects of NiCo@G on the dehydrogenation behavior of LiAlH_4_. Preliminary results showed that excellent dehydrogenation properties of LiAlH_4_ were achieved by 1 wt% NiCo@G doping.

## Results and Discussion

### Preparation and characterization of NiCo@G

**Figure 1:**
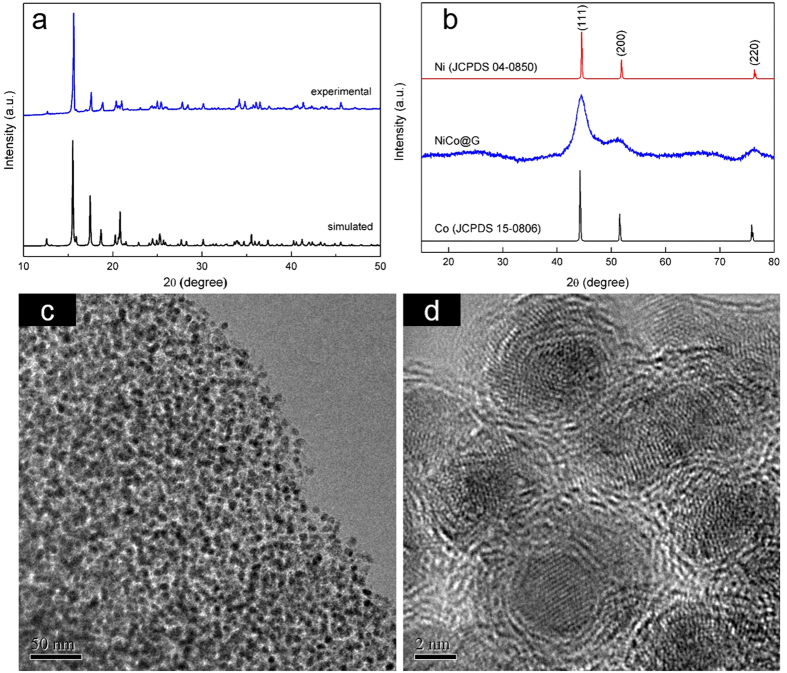
(**a**) XRD patterns of CoCo[Ni(EDTA)]_2_·4H_2_O: simulated pattern on the basis of the single crystal structure according to the already published paper^30^, and experimental pattern of CoCo[Ni(EDTA)]_2_·4H_2_O synthesized in this study; (**b**) XRD patterns of NiCo@G, fcc Ni (JCPDS card no. 04–0850) and fcc Co (JCPDS card no. 15–0806); TEM images of NiCo@G at different magnifications: (**c**,**d**).

Briefly, a 3D bimetallic complex CoCo[Ni(EDTA)]_2_·4H_2_O, a precursor for NiCo@G, was firstly synthesized through solvothermal method. CoCo[Ni(EDTA)]_2_·4H_2_O precursor was thermally decomposed to NiCo@G as the final product at 500 °C in an argon flow. The Powder X-ray diffraction peaks (Fig. 1a) of the as-synthesized precursor CoCo[Ni(EDTA)]_2_·4H_2_O match well with the simulated pattern on the basis of the single crystal structure reported by Sapiña *et al.* (Supplementary Fig. S1)^30^. The Ni/Co molar ratio in NiCo@G is 1:1 as that in the precursor complex, which is confirmed by SEM-EDS data (Supplementary Fig. S2). As shown in Fig. 1b, the XRD pattern of NiCo@G shows peaks at 2*θ* = 44.48, 51.69 and 76.25°, matching those reported for (111), (200) and (220) planes of Ni_50_Co_50_ alloy with a fcc structure^31^,^32^. Their positions are slightly higher than those of pure fcc Co (44.22, 51.52 and 75.86°) and slightly lower than those of pure fcc Ni (44.51, 51.85 and 76.37°). Broadness of the characteristic diffraction peaks for NiCo alloy is due to the formation of nanosized NiCo particles. Furthermore, the characteristic reflections corresponding to hexagonal close packed (hcp) metallic Co can not be observed. The broad peak around 2*θ* = 26° is the characteristic reflection for carbon. X-ray photoelectron spectroscopy (XPS) was used to examine the species present in the particles. The spectra of Ni 2p and Co 2p energy ranges were recorded (Supplementary Fig. S3). The positions of the 2p peaks are respectively 852.8 and 870.1 eV for Ni 2p, 778.3 and 793.3 eV for Co 2p, demonstrating Ni and Co in their zero-valent states^33^,^34^,^35^. TEM images are shown in Fig. 1c,d, indicating that the NiCo nanoalloy is encapsulated in multilayered graphene shells (NiCo@G). The NiCo nanoalloy are spherical in shape, with a highly uniform size distribution ranging from 4 nm to 6 nm. In addition, the STEM corresponding element mapping (Supplementary Fig. S4) of NiCo@G confirms a homogeneous distribution of NiCo alloy over the sample.

### Dehydrogenation performances

**Figure 2:**
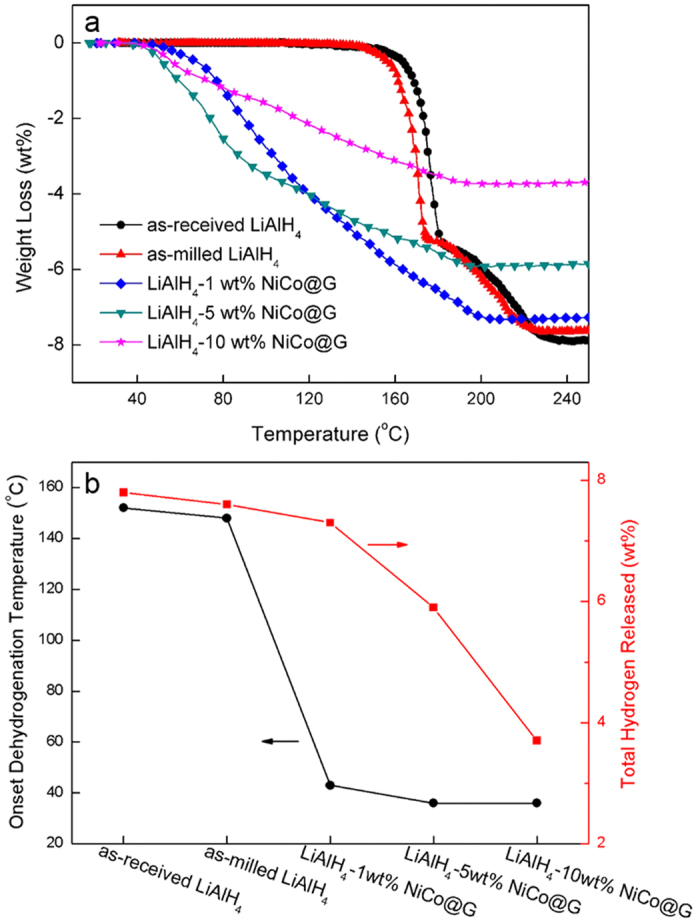
(**a**) Non-isothermal dehydrogenation curves (2 °C min^−1^); (**b**) Onset dehydrogenation temperature and amount of total hydrogen released of as-received LiAlH_4_, as-milled LiAlH_4_ and LiAlH_4_ doped with 1 wt%, 5 wt% and 10 wt% NiCo@G.

Figure 2a as shows the non-isothermal dehydrogenation performances of as-received LiAlH_4_, as-milled LiAlH_4_, and LiAlH_4_ doped with 1 wt%, 5 wt% and 10 wt% NiCo@G. Compared to as-received LiAlH_4_, as-milled LiAlH_4_ exhibits a similar dehydrogenation behavior. The as-received LiAlH_4_ starts to decompose at 152 °C, while the as-milled LiAlH_4_ exhibits a slight decrease of 4 °C. It is obvious that addition of NiCo@G extremely improves the onset dehydrogenation temperature of LiAlH_4_. The onset dehydrogenation temperature and the amount of hydrogen released of all samples are shown in Fig. 2b. The onset desorption temperature decreases with the increasing NiCo@G percent. LiAlH_4_-1 wt% NiCo@G starts to decompose at 43 °C, which is 109 °C lower than as-received LiAlH_4_. For 5 wt% and 10 wt% NiCo@G doped samples, the onset dehydrogenation temperature is as low as 36 °C, which is 116 °C lower than as-received LiAlH_4_. However, raising the NiCo@G percent results in a decrease of the amount of hydrogen released. Only 5.9 wt% and 3.7 wt% of hydrogen are respectively released for LiAlH_4_-5 wt% NiCo@G and LiAlH_4_-10 wt% NiCo@G, due to the increasing catalyst percent and the premature dehydrogenation during the ball milling process. It is noteworthy that the amount of hydrogen released for LiAlH_4_-1 wt% NiCo@G reaches up to 7.3 wt%, which is identical to that of as-milled LiAlH_4_. This phenomenon is attributed to the small NiCo@G percent and a good preservation of hydrogen during the ball milling process. Compared with the performance of other additives or catalysts, NiCo@G developed in this work exhibits high catalytic activity (Table S1^†^). For graphene, Hsu^29^ and Jiang^36^ suggested that the interaction between electronegative carbon and Li^+^, high electronic conductivity promoting electron exchange between metal and [AlH_4_]^−^, and delocalized π bonds facilitates hydrogen release. Furthermore, in NiCo@G, graphene is the shell preventing NiCo nanoparticles aggregation, leading to NiCo nanoalloy with a uniform size distribution ranging from 4 to 6 nm. The beneficial effect of catalyst size on dehydrogenation behaviors has been confirmed in previous literatures.

**Figure 3:**
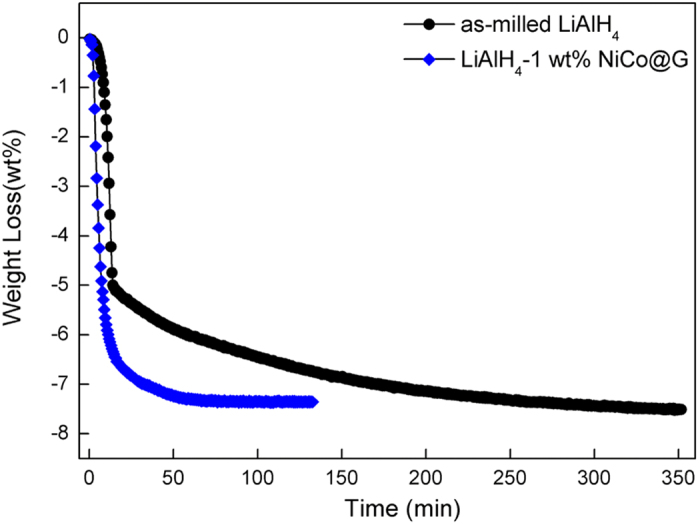
Isothermal dehydrogenation curves of as-milled LiAlH_4_ and LiAlH_4_-1 wt% NiCo@G at 150 °C.

Figure 3 shows the isothermal dehydrogenation kinetics measurements of as-milled LiAlH_4_ and LiAlH_4_-1 wt% NiCo@G at 150 °C. For as-milled LiAlH_4_, only 1.6 wt% of hydrogen releases within 10 min. However, forLiAlH_4_-1 wt% NiCo@G, the dehydrogenation goes on rapidly with 5.8 wt% of hydrogen released within 10 min. Furthermore, total 7.3 wt% of hydrogen can be thoroughly released within 60 min for LiAlH_4_-1 wt% NiCo@G while 350 min for as-milled LiAlH_4_. This result confirms that dehydrogenation kinetics are significantly improved by addition of NiCo@G.

### Dehydrogenation mechanism

**Figure 4:**
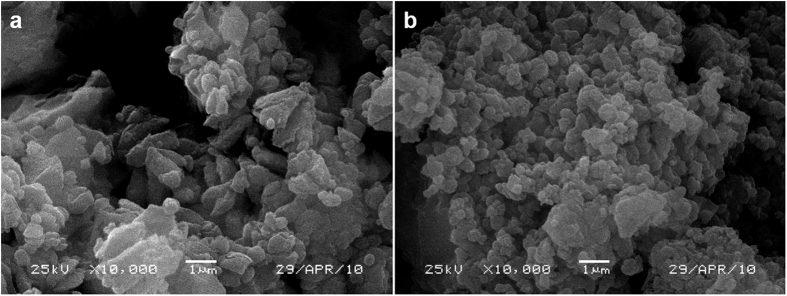
SEM images of: (**a**) as-milled LiAlH_4_, (**b**) LiAlH_4_-1 wt% NiCo@G.

To obtain insight on the catalytic mechanism of NiCo@G for the LiAlH_4_ dehydrogenation, morphologies and intergrain dispersion of both as-milled LiAlH_4_ and LiAlH_4_-1 wt% NiCo@G are investigated by SEM, as shown in Fig. 4. Compared to as-milled LiAlH_4_, the particle size significantly decreases after doping with 1 wt% NiCo@G, leading to more grain boundaries and larger surface area. This important observation suggests that NiCo@G readily influences the LiAlH_4_ texture at room temperature during the ball milling process, by preliminarily breaking their particle aggregation. Graphene has been confirmed as an effective grinding agent to reduce the crystal size of LiAlH_4_ owing to its high mechanical strength^29^,^36^. At that stage it is not yet clear whether the consequent decrease of the dehydrogenation temperature is due to the smaller LiAlH_4_ particles generated after ball milling or to another effect of the catalyst on the mechanism governing this decomposition. The strong catalytic effect of such a small percent of NiCo@G was further investigated by combining XRD and DSC.

**Figure 5:**
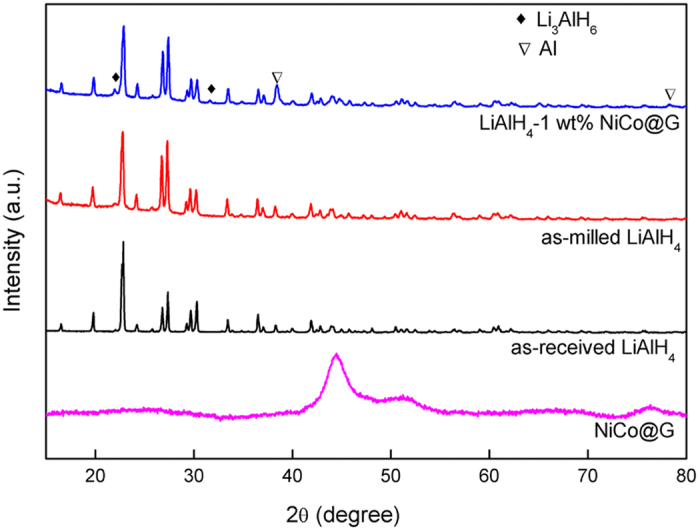
XRD patterns of NiCo@G, as-received LiAlH_4_, as-milled LiAlH_4_ and LiAlH_4_-1 wt% NiCo@G.

Figure 5 shows the XRD patterns of NiCo@G, as-received LiAlH_4_, as-milled LiAlH_4_ and LiAlH_4_-1 wt% NiCo@G. The diffraction peaks of as-milled LiAlH_4_ match well with those of the as-received LiAlH_4_, demonstrating a high stability of LiAlH_4_ during the ball milling process. NiCo@G can not be distinguished in LiAlH_4_-1 wt% NiCo@G, due to the exceptionally small concentration (1 wt%) of NiCo@G. The weak peaks of Li_3_AlH_6_ (2*θ*~21.9°, 31.6°) and Al (111) (2*θ*~38.4°) appear in LiAlH_4_-1 wt% NiCo@G sample, indicating partial dehydrogenation of LiAlH_4_ during ball milling process (R1), in agreement with the small hydrogen capacity loss observed in the TGA visualization of the dehydrogenation process (Fig. 2). Furthermore, the diffraction peaks of LiAlH_4_ in the 1 wt% NiCo@G doped sample become broader than those of as-milled LiAlH_4_, indicating smaller particle size of LiAlH_4_.

**Figure 6:**
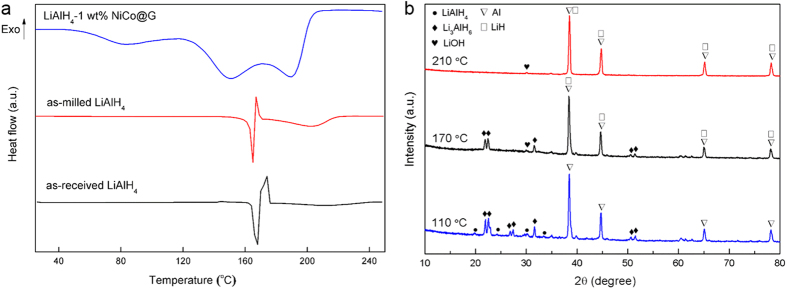
(**a**) DSC profiles of dehydrogenation of as-received LiAlH_4_, as-milled LiAlH_4_ and LiAlH_4_-1 wt% NiCo@G at heating rate of 2 °C min^−1^; (**b**) XRD patterns of LiAlH_4_-1 wt% NiCo@G heated up to different temperatures (110 °C, 170 °C and 210 °C) corresponding to the DSC profile.

DSC measurements were conducted to further verify the effect of NiCo@G on the dehydrogenation of LiAlH_4_, as shown in Fig. 6a. Compared to as-received LiAlH_4_, as-milled LiAlH_4_ presents a similar DSC profile including the melting peak of LiAlH_4_, indicating that the ball milling process does not alter its intrinsic properties. Surprisingly, the DSC profile of LiAlH_4_-1 wt% NiCo@G is totally different and shows three distinct endothermic peaks. In order to understand the phase changes at different stages of LiAlH_4_-1 wt% NiCo@G, we stopped the dehydrogenation of LiAlH_4_-1 wt% at temperatures (110 °C, 170 °C and 210 °C) corresponding to three dehydrogenation stages in DSC profile and investigated the samples by XRD. As shown in Fig. 6b, Li_3_AlH_6_, Al and small amount of retained LiAlH_4_ are present in the sample which was stopped dehydrogenation at 110 °C. Thus, the first endo peak in the DSC profile of LiAlH_4_-1 wt% NiCo@G is attributed to the decomposition of solid LiAlH_4_ (eqn.1). This decomposition (eqn. 1) apparently ends around 110 °C, which explains the absence of any LiAlH_4_ melting. For the sample heated up to 170 °C corresponding to the second stage, Al, LiH, small amount of Li_3_AlH_6_ and LiOH are observed. Al, LiH and small amount of LiOH are present in the sample decomposed at 210 °C. So we can declare that the second and third peaks are attributed to the decomposition of solid Li_3_AlH_6_ (eqn. 2).

### Activation energies

**Figure 7:**
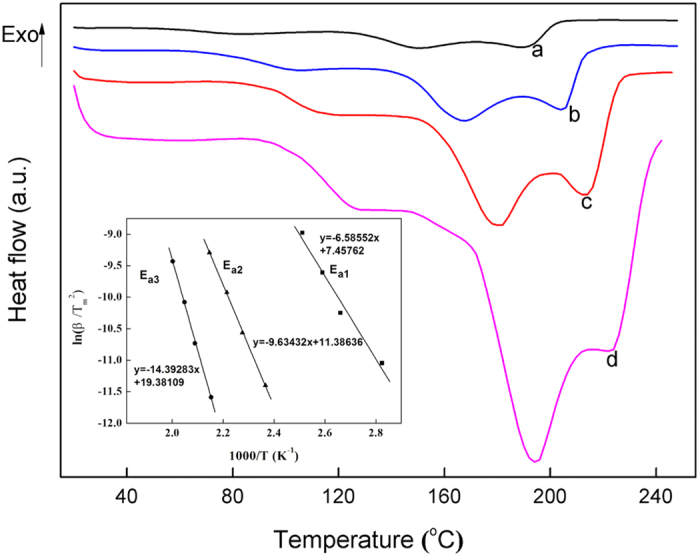
DSC profiles of LiAlH_4_-1 wt% NiCo@G at heating rates of 2 (**a**), 5 (**b**), 10 (**c**) and 20 °C min^−1^ (**d**). The inset graph is Kissinger plots for the three stages of dehydrogenation of LiAlH_4_-1 wt% NiCo@G.

To understand the dehydrogenation kinetics, the apparent activation energy (E_a_) at each stage of LiAlH_4_-1 wt% NiCo@G dehydrogenation was calculated using the Kissinger equation (eqn. 3), considering the three endothermic peaks in DSC profiles at heating rates of 2, 5, 10 and 20 °C min^−1^, as shown in Fig. 7.


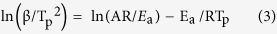


where β is the heating rate, T_p_ (K) is the DSC peak temperature, A is the pre-exponential factor, and R is the gas constant. E_a_, for each dehydrogenation stage of LiAlH_4_-1 wt% NiCo@G, was evaluated to be 54.8 ± 6 kJ mol^−1^, 80.1 ± 1.3 kJ mol^−1^ and 119.7 ± 2.8 kJ mol^−1^ respectively. The activation energy E_a1_ for R1 involved in the catalytic dehydrogenation of LiAlH_4_ is comparable to the lowest values of those reported catalysts.

Regarding the superior efficiency of NiCo@G, many factors obviously play a role. Such efficiency can either be attributed to the support (carbon)^29^ or to a combination of properties associated with two different nanosized metals^37^,^38^ or to both^19^,^39^,^40^. The mechanism need further investigations for the particular NiCo bimetallic nanoparticles encapsulated in graphene used here.

As a summary, NiCo nanoalloy (4–6 nm) encapsulated in grapheme layers (NiCo@G) was prepared and introduced into LiAlH_4_ by solid-state ball milling. A tremendous improvement in the dehydrogenation properties of LiAlH_4_ was achieved. When 1 wt% NiCo@G was doped with LiAlH_4_, the onset dehydrogenation temperature is decreased to 43 °C with 7.3 wt% of hydrogen released below 200 °C. For LiAlH_4_ doped with 10 wt% NiCo@G, the onset dehydrogenation temperature is as low as 36 °C, which is 116 °C lower than that of pristine LiAlH_4_. Ea of LiAlH_4_-1 wt% NiCo@G for the first dehydrogenation step decreased to 54.8 kJ mol^−1^. The significant catalytic effect makes NiCo@G a promising candidate for LiAlH_4_ dehydrogenation. A more in depth study of the effect of NiCo@G on LiAlH_4_ dehydrogenation, in particular regarding the critical roles of Ni/Co and catalyst/LiAlH_4_ ratios, is in process.

## Methods

### Chemicals

All reagents and chemicals were commercially available and of analytical grade without further purification prior to use, unless specifically stated elsewhere.

### Preparationof NiCo@G

The precursor complex CoCo[Ni(EDTA)]_2_·4H_2_O was synthesized by solvothermal method as reported previously^30^. Pyrolysis of CoCo[Ni(EDTA)]_2_·4H_2_O was performed under an argon (99.999%) flow at 500 °C for 3 h. The final sample was denoted as NiCo@G.

### Preparation of LiAlH_4_-NiCo@G samples

LiAlH_4_ (97%) was purchased from Alfa Aesar, and used without further purification. Typically, 0.5 g powder mixture composed of LiAlH_4_ and NiCo@G was loaded into a stainless milling pot with 10 steel balls (10 mm in diameter). Ball milling was carried out on a QM-1SP2 planetary under an argon atmosphere at 300 rpm for 30 min. All sample handlings were performed in a glove box filled with argon to avoid oxidation and moisture.

### Characterizations

Powder X-ray diffraction (XRD) measurements were conducted on a PANalytical X’pert diffractometer operated at 40 kV and 40 mA with a Cu K_α_ radiation (*λ* = 1.5418 nm). The samples were covered by Mylar film in glove box to avoid oxidation and moisture. Scanning electron microscopy (SEM) images were obtained by using JSM-6360LV SEM (JEOL, Japan). Transmission electron microscopy (TEM) studies were performed on a FEI Tecnai F30 microscope and a G^2^ microscope operated at 300 kV. The powders were dropped on an ultrathin carbon film supported on a copper grid by using ethanol as a dispersant. X-ray photoelectron spectroscopy (XPS) was recorded using a Thermo ESCALAB 250Xi instrument with Al Kα X-rays (1486.6 eV). Thermogravimetric analysis (TGA) was carried out on a Cahn Thermax 500 with a heating rate of 2 °C min^−1^ in an argon flow. The isothermal dehydrogenation kinetics were measured using a Sieverts-type apparatus (Advanced Materials Corporation, USA) at 150 °C under an initial pressure of 10^−5^ MPa. Differential scanning calorimetry (DSC) data was collected from a TA Q1000 in a constant argon flow (50 mL min^−1^) at different heating rates (2 K min^−1^, 5 K min^−1^,10 K min^−1^, 20 K min^−1^).

## Additional Information

**How to cite this article**: Jiao, C. *et al.* NiCo nanoalloy encapsulated in graphene layers for improving hydrogen storage properties of LiAlH_4_. *Sci. Rep.* **6**, 27429; doi: 10.1038/srep27429 (2016).

## Supplementary Material

Supplementary Information
