# Supplementary material for: NiCo nanoalloy encapsulated in graphene layers for improving hydrogen storage properties of LiAlH4
Source: Sci Rep. 2016 Jun 7;6:27429. doi: 10.1038/srep27429 (PMC4895212; doi:10.1038/srep27429)
Supplement: Supplementary Information [file srep27429-s1.doc]

Supplementary Information

**NiCo nanoalloy encapsulated in graphene layers for improving hydrogen storage properties of LiAlH_4_**

Chengli Jiao^1, 2, 4^, Lixian Sun^2, *^, Fen Xu^2, *^, Shu-Sheng Liu^3^, Jian Zhang^4^, Xia Jiang^4^, & Lini Yang^5^

^1^Key Laboratory of Biobased Materials, Qingdao Institute of Bioenergy and Bioprocess Technology, Chinese Academy of Sciences, No.189 Songling Road, Qingdao 266101, P.R. China

^2^Guangxi Key Laboratory of Information Materials & Guangxi Collaborative Innovation Center of Structure and Property for New Energy and Materials, School of Material Science and Engineering, Guilin University of Electronic Technology, Guilin 541004, P.R. China

^3^INAMORI Frontier Research Center, Kyushu University, Nishi-ku, Fukuoka 8190395, Japan

^4^Dalian Institute of Chemical Physics, Chinese Academy of Sciences, 457 Zhongshan Road, Dalian 116023, P.R. China

^5^College of Chemistry, Liaoning University, Shenyang 110036, P.R. China

^*^ Corresponding authors:

Lixian Sun, E-mail: [sunlx@guet.edu.cn](mailto:sunlx@guet.edu.cn); Fen Xu, E-mail: [xufen@guet.edu.cn](mailto:xufen@guet.edu.cn), Tel: +86-773-2303763


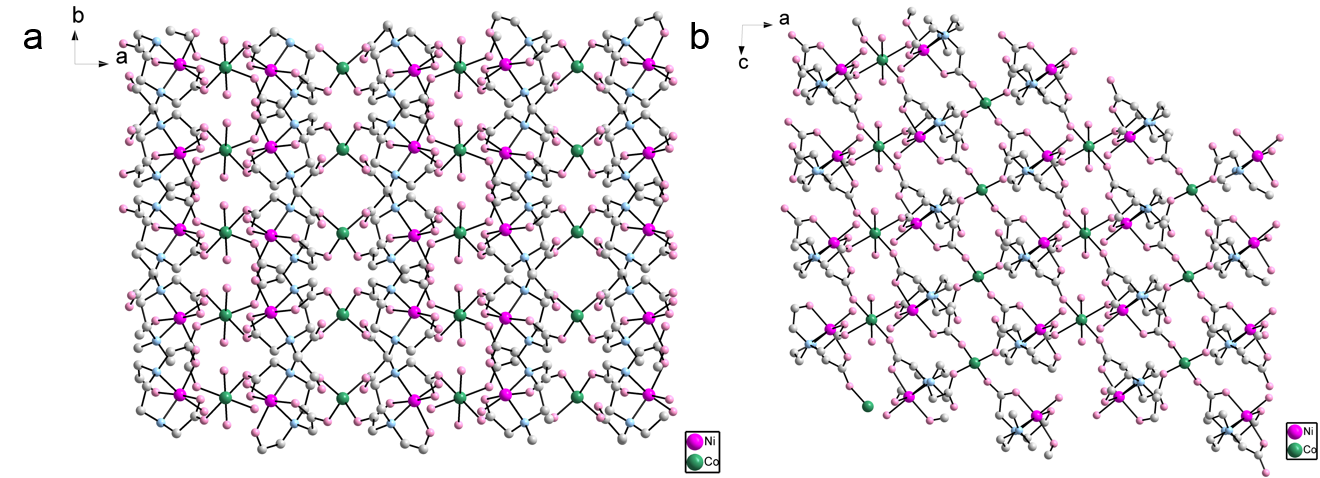


**Figure S1.** Crystal structure of CoCo[Ni(EDTA)]_2_·4H_2_O^1^: (a) packing diagram along [001]; (b) packing diagram along [010].


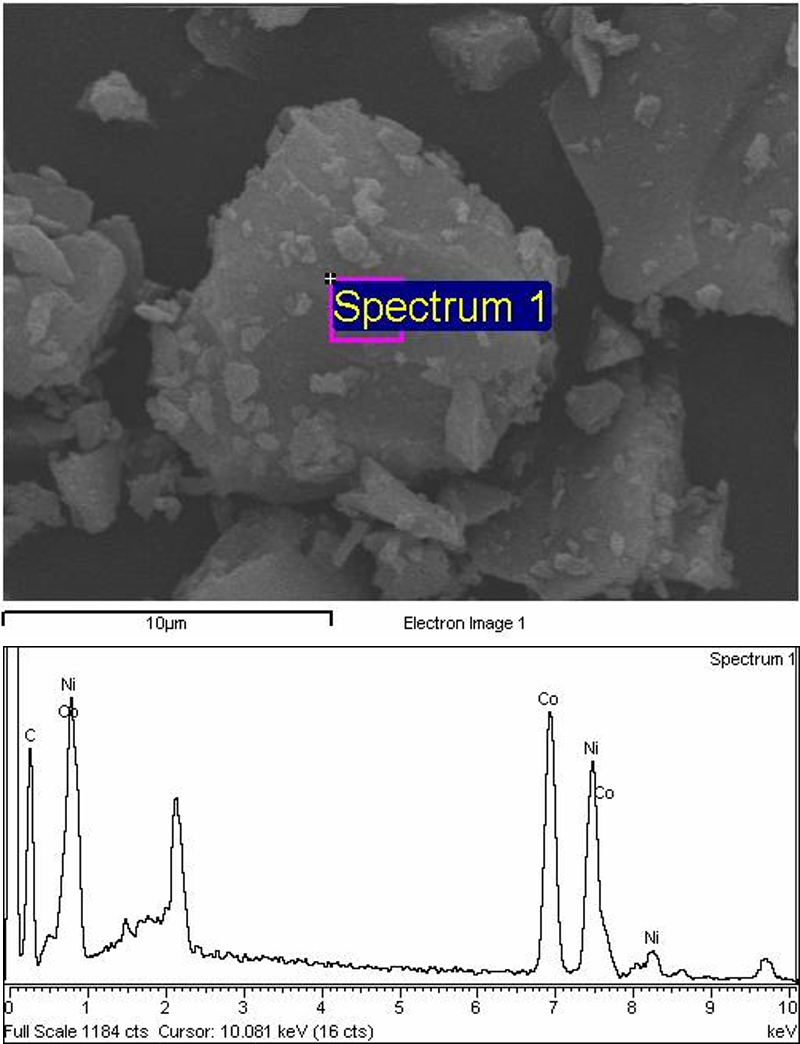


**Figure S2.** SEM image and EDS profile of NiCo@G.


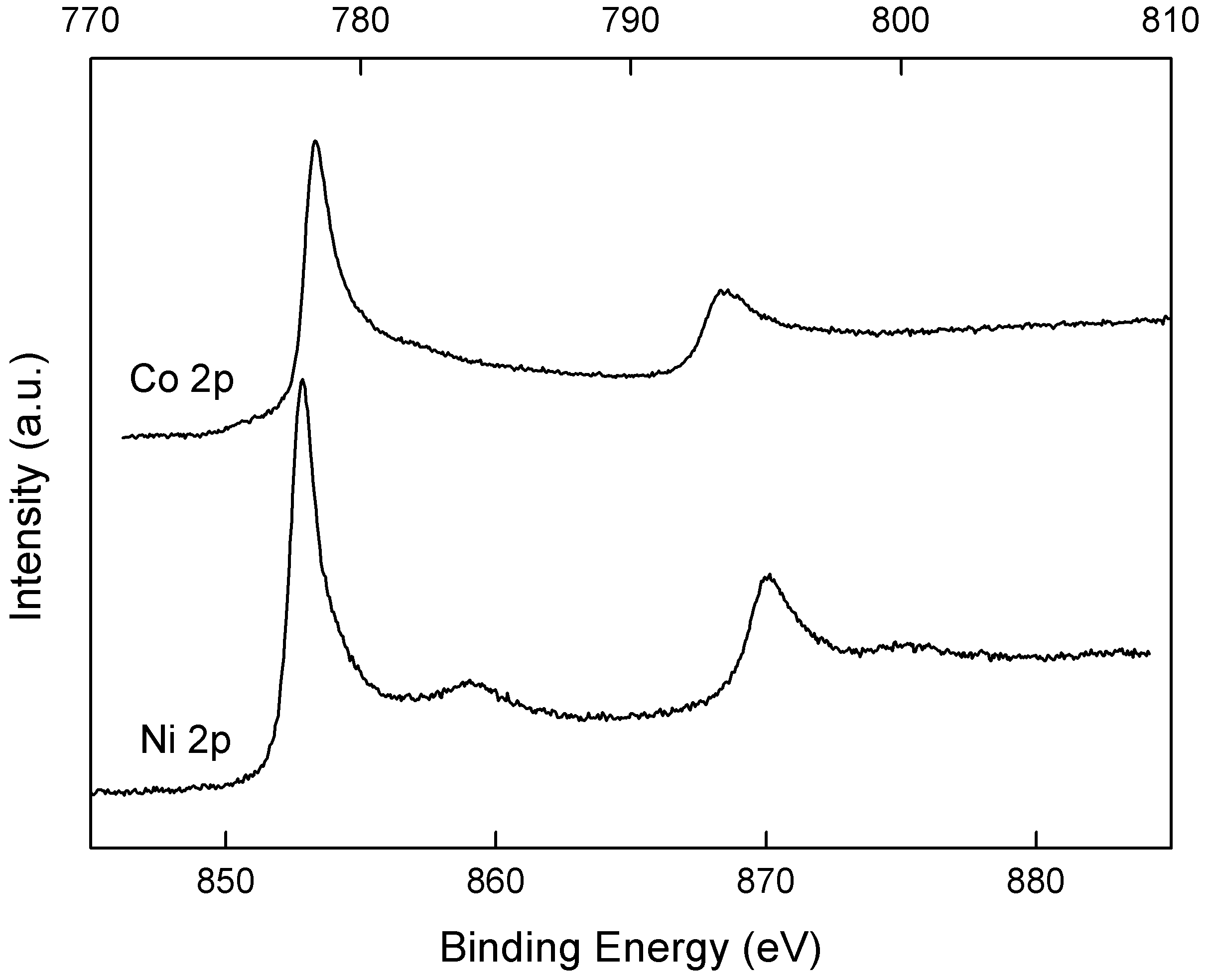


**Figure S3.** Ni 2p and Co 2p XPS of NiCo@G.


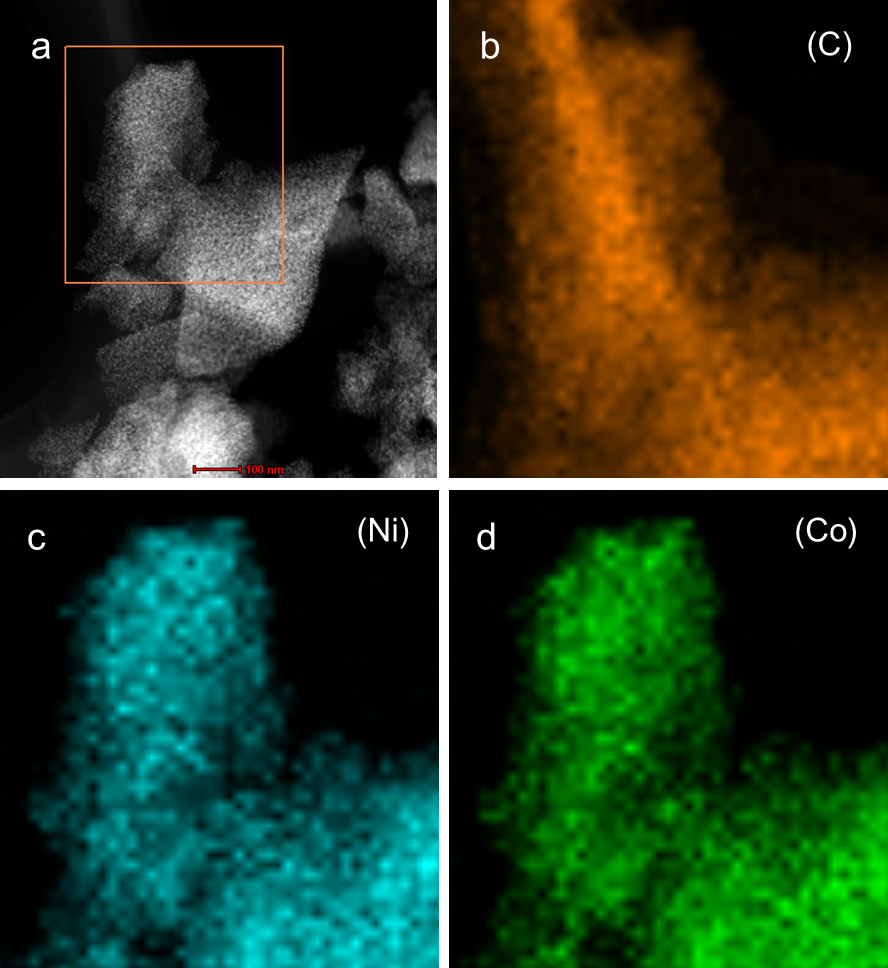


**Figure S4.** (a) STEM image of NiCo@G; EDX mapping images of (b) carbon, (c) nickel and (d) cobalt.

**Table S1** Comparison of the performance of NiCo@G with other additives from literatures^2-13^.

| **Additive/Catalyst** | **Doping content** | **Onset temp. (°C)** | **Total amount of H_2_ released (wt%)** | **Reference** |
| --- | --- | --- | --- | --- |
| Ni | 5 mol% | 145 | 5.1 | 2 |
| NiFe_2_O_4_ | 3 mol% | 61 | 7.2 | 3 |
| NiCo_2_O_4_ | 2 mol% | 141 | 7.1 | 4 |
| CoFe_2_O_4_ | 5 mol% | 100 | 3.2 | 5 |
| Ti | 5 mol% | 135 | 5.1 | 2 |
| TiF_3_ | 4 mol% | 80 | 6.3 | 6 |
| Ce(SO_4_)_2_ | 5 mol% | 110 | 6.1 | 2 |
| SWCNT | 5 wt% | 80 | 7.2 | 7 |
| MWCNTs/0.1Ni | 20 wt% | 102 | 4.6 | 8 |
| MWCNTs/0.4Ni | 20 wt% | 77 | 4.3 | 8 |
| MWCNTs/0.1Co | 20 wt% | 138 | 4.7 | 8 |
| MWCNTs/0.4Co | 20 wt% | 103 | 4.0 | 8 |
| MWCNTs/Pt | 20 wt% | 156 | 5.1 | 9 |
| MWCNTs/Pd | 20 wt% | 143 | 5.1 | 9 |
| TiCl_3_/SWCNT | 5 wt% | 102 | 6.2 | 10 |
| Graphene | 15 wt% | 80 | 6.4 | 11 |
| Fe/Graphene nanosheet | 2.5 wt% | 40 | - | 12 |
| Ni/Graphene nanosheet | 2.5 wt% | 60 | - | 12 |
| Co@C | 10 wt% | 100 | 7.05 | 13 |
| NiCo@G | 1 wt% | 43 | 7.3 | This work |
| NiCo@G | 5 wt% | 36 | 5.9 | This work |

**References**

1. Sapina, F., Coronado, E., Beltran, D. & Burriel, R. From 1-D to 3-D ferrimagnets in the EDTA family - magnetic characterization of the tetrahydrate series MtM(M'EDTA)_2_.cntdot.4H_2_O [Mt, M, M' = cobalt(II), nickel(II), zinc(II)]. *J. Am. Chem. Soc.* **113**, 7940-7944 (1991).

2. Zheng, X. & Liu, S. Study on hydrogen storage properties of LiAlH_4_. *J. Alloys Compd.* **481**, 761-763 (2009).

3. Li, P. et al. NiFe_2_O_4_ nanoparticles catalytic effects of improving LiAlH_4_ dehydrogenation properties. *J. Phys. Chem. C* **117**, 25917-25925 (2013).

4. Li, L. et al. Enhancement of the H_2_ desorption properties of LiAlH_4_ doping with NiCo_2_O_4_ nanorods. *Int. J. Hydrogen Energy* **39**, 4414-4420 (2014).

5. Li, Z. et al. Enhanced hydrogen storage properties of LiAlH_4_ catalyzed by CoFe_2_O_4_ nanoparticles. *RSC Adv.* **4**, 18989-18997 (2014).

6. Liu, S. S. et al. Effect of ball milling time on the hydrogen storage properties of TiF_3_-doped LiAlH_4_. *Int. J. Hydrogen Energy* **34**, 8079-8085 (2009).

7. Ismail, M., Zhao, Y., Yu, X. B., Ranjbar, A. & Dou, S. X. Improved hydrogen desorption in lithium alanate by addition of SWCNT-metallic catalyst composite. *Int. J. Hydrogen Energy* **36**, 3593-3599 (2011).

8. Tan, C. Y. & Tsai, W. T. Effects of Ni and Co-decorated MWCNTs addition on the dehydrogenation behavior and stability of LiAlH_4_. *Int. J. Hydrogen Energy* **40**, 14064-14071, (2015).

9. Tan, C. Y. & Tsai, W. T. Catalytic and inhibitive effects of Pd and Pt decorated MWCHTs on the dehydrogenation behavior of LiAlH_4_. *Int. J. Hydrogen Energy* **40**, 10185-10193, (2015).

10. Tan, C. Y. & Tsai, W. T. Effects of TiCl_3_-decorated MWCNTs addition on the dehydrogenation behavior and stability of LiAlH_4_. *Int. J. Hydrogen Energy* **39**, 20038-20044, (2014).

11. Hsu, C. P. et al. Buckyball-, carbon nanotube-, graphite-, and graphene-enhanced dehydrogenation of lithium aluminum hydride. *Chem. Commun.* **49**, 8845-8847 (2013).

12. Jiang, D. H., Yang, C. H., Tseng, C. M., Lee, S. L. & Chang, J. K. Metal/graphene nanocomposites synthesized with the aid of supercritical fluid for promoting hydrogen release from complex hydrides. *Nanoscale* **6**, 12565-12572 (2014).

13. Li, L., Wang, Y., Jiao, L. & Yuan, H. Enhanced catalytic effects of Co@C additive on dehydrogenation properties of LiAlH_4_. *J. Alloys Compd.* **645**, S468-S471, (2015).
